# Long-term implant survival after debridement, antibiotics and implant Retention (DAIR) for acute prosthetic joint infections: is it a viable option beyond four weeks after index arthroplasty?

**DOI:** 10.1007/s00264-025-06422-6

**Published:** 2025-02-14

**Authors:** Juan Carlos Perdomo-Lizarraga, Andrés Combalia, Jenaro Ángel Fernández-Valencia, Juan Carlos Martínez-Pastor, Laura Morata, Alex Soriano, Ernesto Muñoz-Mahamud

**Affiliations:** 1 Department of Orthopaedics and Trauma Surgery, Hospital Clínic of Barcelona. University of Barcelona. Barcelona (UB), Spain. c. Villarroel, 170, 08036 Barcelona, Spain, Barcelona, Spain; 2 Departament de Cirurgia i Especialitats Medicoquirúrgiques, Facultat de Medicina i Ciències de la Salut, Universitat de Barcelona (UB), c. Casanova, 143, 08036 Barcelona, Spain., Barcelona, Spain; 3 Department of Infectious Diseases, Hospital Clínic of Barcelona. University of Barcelona. Barcelona, Spain., Barcelona, Spain; 4 Institut d’Investigacions Biomèdiques August Pi i Sunyer (IDIBAPS), c. Villarroel, 170, 08036 Barcelona, Spain., Barcelona, Spain

**Keywords:** Prosthetic joint infection, Debridement, Hip arthroplasty, Knee arthroplasty, Long-term follow-up

## Abstract

**Purpose:**

Debridement, Antibiotic Treatment, and Implant Retention (DAIR) is considered the first-line treatment for early acute Prosthetic Joint Infection (PJI). This study aims to evaluate the five year success rates of early acute PJI managed with DAIR taking into consideration the time from the index surgery.

**Materials and methods:**

A retrospective analysis of medical charts for 291 consecutive patients with acute PJI occurring within the first three months after primary or revision arthroplasty was conducted. Patients were stratified into two groups based on DAIR timing: Group (A) patients who underwent DAIR within the first four weeks post-arthroplasty; Group (B) patients who underwent DAIR between five and 12 weeks post- arthroplasty. Success rate was defined as implant in place, without signs of infection and not under suppressive antibiotic treatment.

**Results:**

The overall five year success rate for the entire cohort at five years was 62.2%. The mortality rate during the study period was 8.2%. The five year success rate was 64.4% (141 of 219) for Group A and 55.6% (40 of 72) for Group B (*p* = 0.21). Including deceased patients without signs of infection and retained implants as successful cases, the five year success rates increased to 69.9% for Group A (153 out of 219) and 69.4% for Group B (50 out of 72). The implant survival rate at five years was 73% for Group A and 71% for Group B.

**Conclusion:**

Our findings indicate that there are no significant differences between patients who undergo a DAIR procedure within four weeks from those performed between week five and 12. Importantly, the overall success rate decreased from 75.6 to 62.2% in the last three years of follow-up.

## Introduction

Acute prosthetic joint infection (PJI) is a severe complication of joint arthroplasty, with incidences reported between 1 and 2% after primary surgery and 3–10% following revision surgery [1–3]. For managing early acute PJI, Debridement, Antibiotic Therapy, and Implant Retention (DAIR) is often the first-line approach, offering benefits such as reduced surgical complexity, quicker recovery, bone stock preservation, favorable long-term functional outcomes, and cost-efficiency compared to implant revision [4–7].

However, the optimal timing for DAIR in treating acute PJI remains uncertain. Some guidelines, such as those from the Infectious Diseases Society of America (IDSA) published in 2013, recommend DAIR only for infections identified within the first month post-surgery [8]. Nevertheless, other studies suggest that while DAIR success rates may decline when performed beyond four weeks, acceptable outcomes are still achievable [9]. The primary limitation of these studies is the short follow-up period, typically restricted to one year post-DAIR. This study aims to address this gap by investigating the impact of DAIR timing on long-term implant retention rates.

The objective of this study was to assess five year success rates of DAIR in treating postoperative acute PJI based on timing from the index surgery. This research seeks to inform long-term outcomes of DAIR in early acute PJI management, enhancing treatment strategies for such cases.

## Materials and methods

### Study design

This study involved a retrospective analysis of medical charts for all consecutive patients presenting with postoperative acute PJI after primary or revision hip or knee arthroplasty performed between 1st January of 1999 and 31st March of 2018. The inclusion criteria included patients undergoing DAIR for an acute PJI occurring within the first 3 months following the index arthroplasty, with a minimum follow-up period of five years established as a cut-off. Only patients with at least five years of follow-up were included in the study.

Patients were stratified into two groups based on DAIR timing: Group (A) patients who underwent DAIR within the first four weeks post-arthroplasty; Group (B) patients who underwent DAIR between five and 12 weeks post- arthroplasty.

### Definitions and variables

**Fig. 1:**
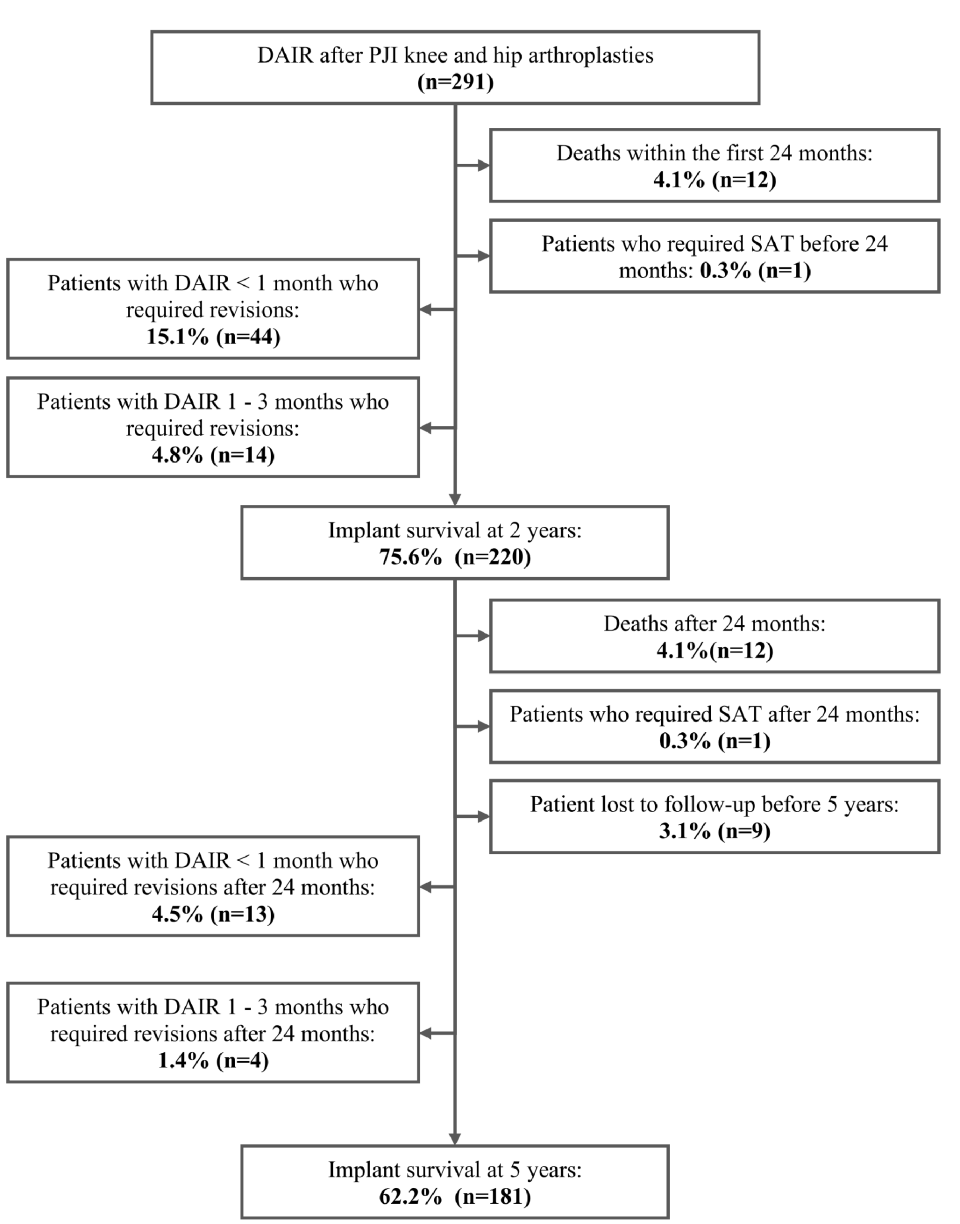
Patient inclusion flowchart. Failure was defined as the need for suppressive antibiotic therapy or any-cause revision. SAT: suppressive antibiotic therapy. PJI: Prosthetic joint infection

Acute PJI was defined as clinical signs and symptoms of infection lasting ≤ three weeks and occurring within 12 weeks of the index arthroplasty. Diagnosis was based on the 2013 International Consensus Meeting criteria [10]. Five-year success was defined by implant retention without infection signs and without suppressive antibiotic therapy (SAT), with patients alive at follow-up. Figure 1 depicts the patient inclusion process. Variables analyzed included joint type (hip or knee), surgery indication (primary or revision arthroplasty), revision type (septic or non-septic), isolated microorganisms, and mortality rate.

### Ethical requirements

Data for this study were retrospectively extracted and anonymized. This study was reviewed and approved by the Ethical Institutional Review Board of the hospital with the code HCB/2023 − 0492.

### Statistical analysis

Continuous variables are reported as means and standard deviations (SD), while categorical variables are presented as counts and percentages. Statistical significance was assessed using the Chi-square test for categorical variables and Student’s t-test for continuous variables. Kaplan-Meier survival curves with 95% confidence intervals were constructed, and the Log-Rank test was employed to compare failure probability based on DAIR timing relative to primary arthroplasty. Data were anonymized and stored in Excel, and analyses were conducted using IBM SPSS 29.0.0 (SPSS, Inc., Chicago, IL).

## Results

A total of 291 patients were included in the final analysis: Group A comprised 219 patients, while Group B consisted of 72 patients. The main demographic and clinical characteristics of the cohort are summarized in Table 1. No significant differences were observed between the two groups in terms of age, sex, BMI, infection location, type of index surgery, or comorbidities. However, monomicrobial infections were more prevalent in Group A (62.1% vs. 47.2%, *p* = 0.02), while culture-negative PJI was more frequent in Group B (10.5% vs. 27.8%, *p* < 0.001).

**Table 1:** Main demographics of the patients included in the study

Variable	All patients *n* = 291 (100%)		Group A (1–4 weeks) *n* = 219 (75.3%)			Group B (5–12 weeks) *n* = 72 (24.7%)			*p*-value
	*n*	%	*n*		%	*n*		%	
**Mean Age (SD)**	70.6	(10.9)	70.7		(11.1)	70.4		(10.2)	0.86
**Sex**									
Female	160	55	121		55.3	39		54.2	0.87
Male	131	45	98		44.7	33		45.8	
**Joint**									
Knee	175	60.1	126		57.5	49		68.1	0.11
Hip	116	39.9	93		42.5	23		31.9	
**Type of surgery**									
Primary THA	228	78.4	175		79.9	53		73.6	0.26
Revision	63	21.6	44		20.1	19		26.4	
**ASA Classification**									
I	25	8.6	17		7.8	8		11.1	0.37
II	153	52.6	121		55.3	32		44.4	0.11
III-IV	86	29.6	59		26.9	27		37.5	0.08
N/A	27	9.2	22		10	5		7	0.43
**Comorbidities**									
No comorbidity	48	16.6	35		16	13		18.1	0.68
Diabetes mellitus	51	17.5	36		16.4	15		20.8	0.39
Malignancy	26	8.9	20		9.1	6		8.3	0.83
Renal disease	27	9.3	21		9.6	6		8.3	0.75
Liver cirrhosis	19	6.5	14		6.4	5		7	0.86
HIV infection	3	1	1		0.5	2		2.8	0.09
Rheumatic disease	14	4.8	8		3.7	6		8.3	0.10
Smoking	31	10.7	20		9.1	11		15.3	0.18
**Microbiology**									
Monomicrobial	170	58.4	136		62.1	34		47.2	**0.02**
Polymicrobial	77	26.5	59		26.9	18		25	0.74
Negative cultures	43	14.8	23		10.5	20		27.8	**< 0.001**
N/A	1	0.3	1		0.5	0		0	0.56

SD: standard deviations; ASA: American Society of Anesthesiology; N/A: not available; HIV: human immunodeficiency virus. THA: total hip arthroplasty

Although the distribution of isolated microorganisms showed no statistically significant differences between the groups, coagulase-negative staphylococci (CoNS) was the predominant pathogen in Group A (37.9%) and the second most common in Group B (30.5%). Staphylococcus aureus was the second most common pathogen in Group A (26.5%) and the most frequent in Group B (30.6%) (Table 2).

**Table 2:** Summary of microbiological report of intraoperative cultures obtained during the debridement

Variable	All patients *n* = 291 (100%)		Group A (1–4 weeks) *n* = 219 (75.3%)		Group B (5–12 weeks) *n* = 72 (24.7%)		*p*-value
	*n*	%	*n*	%	*n*	%	
**Gram positive cocci**							
Coagulase-negative staphylococci	105	36.1	83	37.9	22	30.5	0.32
*Staphylococcus aureus*	70	24.1	58	26.5	22	30.6	0.50
*Enterococcus faecalis*	25	8.6	20	9.1	5	6.9	0.56
Group viridans streptococci	3	1	1	0.5	2	2.8	0.15
*Streptococcus agalactiae*	2	0.7	2	0.9	0	0	0.41
*Corynebacterium* spp	2	0.7	0	0	2	2.8	0.06
*Enterococcus faecium*	1	0.3	1	0.5	0	0	0.56
**Gram negative bacilli**							
*Pseudomonas aeruginosa*	30	10.3	26	11.9	4	5.6	0.12
*Escherichia coli*	22	7.6	18	8.2	4	5.6	0.45
*Proteus mirabilis*	14	4.8	11	5	3	4.2	0.76
*Enterobacter cloacae*	13	4.5	11	5	2	2.8	0.42
*Klebsiella pneumoniae*	11	3.8	10	4.6	1	1.4	0.22
*Morganella morganii*	4	1.4	2	0.9	2	2.8	0.23
*Serratia marcescens*	3	1	2	0.9	1	1.4	0.72
*Citrobacter koseri*	3	1	3	1.4	0	0	0.31
*Enterobacter gergoviae*	1	0.3	1	0.5	0	0	0.56
*Acinetobacter baumanii*	1	0.3	1	0.5	0	0	0.56
*Ralstonia pickettii*	1	0.3	1	0.5	0	0	0.56
*Campylobacter fetus*	1	0.3	1	0.5	0	0	0.56
**Anaerobes**							
*Peptostreptococcus* spp	2	0.7	0	0	2	2.8	**0.01**
*Bacteroides fragilis*	1	0.3	1	0.5	0	0	0.56
*Rothia dentocariosa*	1	0.3	1	0.5	0	0	0.56
**Fungi**							
*Candida parapsilosis*	1	0.3	1	0.5	1	1.4	0.40

For the entire cohort, the overall success rate at five years (implant survival without signs of infection or the need for suppressive antibiotic therapy) was 62.2% (181 out of 291). The overall success rate decreased from 75.6 to 62.2% in the last three years of follow-up. The mortality rate over the study period was 8.2% (24 out of 291). Excluding two patients who died with active infection, the remaining 22 deceased patients did not require implant removal and were considered infection-free at the time of death. When these patients were included as successful cases, the adjusted success rate at five years was 70.1% (204 out of 291).

**Fig. 2:**
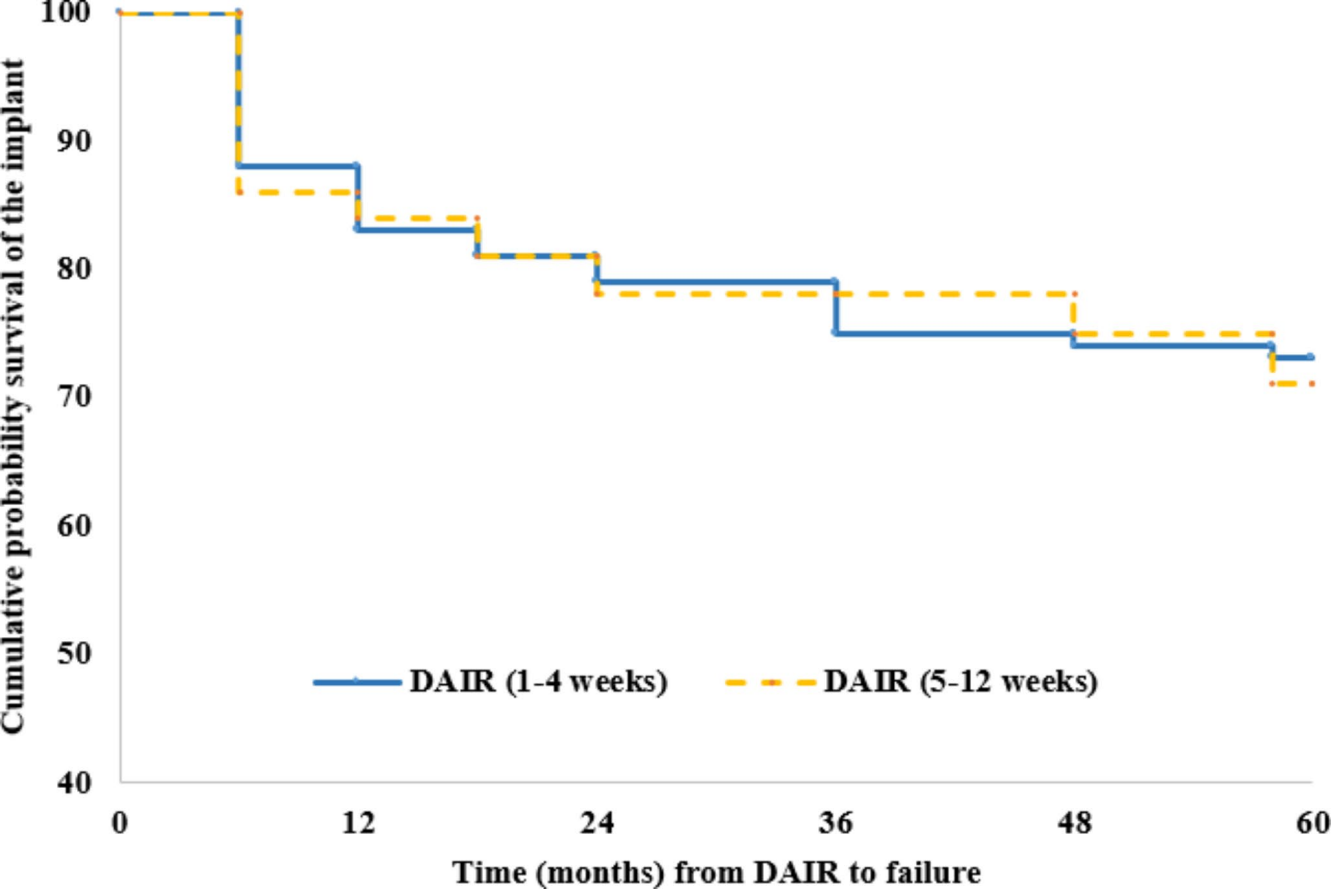
Kaplan-Meier survival curve showing no significant difference in the survival probability according to the time from DAIR (Debridement, Antibiotics, and Implant Retention) to failure in 5 years of follow-up

The five year success rate was 64.4% (141 of 219) for Group A and 55.6% (40 of 72) for Group B (*p* = 0.21) (Table 3). Including deceased patients without signs of infection and retained implants as successful cases, the five year success rates increased to 69.9% for Group A (153 out of 219) and 69.4% for Group B (50 out of 72). The Kaplan-Meier estimated implant survival rate at five years for each group is shown in Fig. 2.

**Table 3:** Outcomes at 5 years of follow-up

Variable	All patients *n* = 291 (100%)		Group A (1–4 weeks) *n* = 219 (75.3%)		Group B (5–12 weeks) *n* = 72 (24.7%)		*p*-value
	*n*	%	*n*	%	*n*	%	
Deaths	24	8.2	13	5.9	11	15.3	**0.01**
Loss of follow-up*	9	3.1	7	3.2	2	2.8	0.85
SAT	2	0.7	1	0.5	1	1.4	0.40
Revisions at 5 years	75	25.8	57	26	18	25	0.86
Implant survival at 5 years	181	62.2	141	64.4	40	55.6	0.21
**Reasons for revision**							
Septic	55	18.9	44	20.1	11	15.3	0.17
Non septic	20	6.9	13	5.9	7	9.7	
**Microbiology at revision**							
Monomicrobial	34	11.7	24	11	10	13.9	0.31
Polymicrobial	10	3.4	8	5	2	2.8	0.75
Negative	29	10	23	10.5	6	8.3	0.59
N/A	2	0.7	2	0.9	0	0	0.42
**Strategy for septic revision**							
1-Stage revision	7	2.4	6	2.7	1	1.4	0.68
2-Stage revision	48	16.6	38	17.4	10	13.9	

SAT: suppressive antibiotic treatment; *Loss of follow-up between 3 to 5 years after DAIR; N/A: not available

No differences in implant survival were found based on the affected joint. Among the 291 patients, 116 had hip arthroplasties and 175 had knee arthroplasties. The five year implant survival rate was 58.6% for hip arthroplasties (68 of 116) and 64.6% for knee arthroplasties (113 of 175) (*p* = 0.30).

## Discussion

While DAIR is widely accepted as the preferred treatment strategy for postoperative acute PJI, there is ongoing debate regarding the optimal time frame within which it can be performed successfully. The present study aimed to evaluate the five year success rates of DAIR for acute PJIs occurring within different time intervals following index arthroplasty. Results indicated no significant difference in outcomes between patients who underwent DAIR within the first four weeks versus those treated between weeks five and 12.

These findings align with a recent multi-centre study that also reported no significant differences in revision rates at one year follow-up across similar time frames [9]. In that study, which included 769 patients, the one year failure rate reached 38%, with failure rates of 42% for weeks one to two, 38% for weeks three to four, 29% for weeks five to six, and 42% for weeks seven to12. Similarities in causative microorganisms, comorbidities, and symptom duration were observed across time intervals, supporting the viability of DAIR beyond the four week mark, provided it is performed within one week of symptom onset and modular component exchange is feasible.

Conversely, current IDSA guidelines recommend DAIR only for PJIs diagnosed within the first four weeks post-index arthroplasty [8]. This limitation potentially excludes a considerable subset of patients who could benefit from DAIR, possibly exposing them to complications related to early implant removal. This consideration is particularly relevant as approximately 25% of early PJIs of the present series manifested between weeks five and 12 after the index surgery.

Other studies, however, present conflicting perspectives. A recent retrospective analysis of 189 knee PJIs managed with DAIR reported that time from index arthroplasty was a significant predictor of outcome, reporting two year success rates of 67.4%, 52.9%, 42.4%, 41.5%, and 29.1% for PJIs treated within zero to one, one to three, three to 12 months, one to three years, and over three years, respectively [11]. These authors concluded that DAIR remains a reasonable option for PJIs occurring within the first year, given the risks associated with staged revision surgery.

Long-term data on DAIR success beyond two years are scarce. Clauss et al. [12] followed 56 patients with hip PJI treated with DAIR, reporting a 16% revision rate after an average follow-up of 6.1 years. Our study observed a higher five year revision rate of 25.8% (75 out of 291), which may reflect differences in patient populations or treatment protocols. Similarly, Grammatopoulos et al. [13] reported an 85% implant survival rate at five years in 122 PJI cases managed with DAIR for both primary and revision hip arthroplasties, with 23% requiring additional surgery. Notably, in that study, prolonged antibiotic therapy lasting from 0.5 to three years was common, and suppressive antibiotic therapy (SAT) was not counted as a failure outcome.

The present study has several inherent limitations. Foremost, its retrospective design may have introduced selection bias, limiting control over confounding variables and constraining the scope of the data collected. Additionally, the absence of a control group is a limitation, and it is important to acknowledge that clinical practices and protocols may have evolved over the study period, potentially affecting outcomes. Consequently, these factors should be considered when interpreting the generalizability of our findings. Nonetheless, the study cohort represents one of the largest published to date. Despite a debatable five year success rate of 70%, we believe that DAIR remains a viable approach for managing acute PJIs within the first three months post-arthroplasty.

In conclusion, our data suggest no significant difference in outcomes between patients undergoing DAIR within four weeks and those treated between weeks five and 12. However, it is noticeable that the overall success rate decreased from 75.6 to 62.2% in the last three years of follow-up. Further prospective studies with larger cohorts and extended follow-up are needed to confirm these findings and refine criteria for DAIR success.

## Data Availability

No datasets were generated or analysed during the current study.

## Declarations

### AI tools use

GPT-3.5 was used for the execution of this article solely as a language corrector.
